# Retraction: The prognostic potential and carcinogenesis of long non-coding RNA TUG1 in human cholangiocarcinoma

**DOI:** 10.18632/oncotarget.28418

**Published:** 2023-08-30

**Authors:** Yi Xu, Kaiming Leng, Zhenglong Li, Fumin Zhang, Xiangyu Zhong, Pengcheng Kang, Xingming Jiang, Yunfu Cui

**Affiliations:** ^1^Department of Hepatopancreatobiliary Surgery, Second Affiliated Hospital of Harbin Medical University, Harbin, China; ^2^Department of The Key Laboratory of Myocardial Ischemia, Harbin Medical University, Ministry of Education, Harbin, China

**This article has been retracted:** Several images in this paper are duplicates of images in other published papers. In Figure 5C, a panel is duplicated within Figure 6D of another paper [1]. In Figure 5B, a panel is duplicated within Figure 2F of another paper [2]. In Figure 6A, a panel is duplicated within Figure 8A of this same paper [2]. Although this article was published earlier than the other articles, the Oncotarget Scientific Integrity Office decided to retract this article given concerns about the accuracy and authenticity of the data. All authors have agreed to this retraction.

Original article: Oncotarget. 2017; 8:65823–65835. 65823-65835. https://doi.org/10.18632/oncotarget.19502
